# Author Correction: Bacterial adaptation is constrained in complex communities

**DOI:** 10.1038/s41467-021-27581-1

**Published:** 2021-12-15

**Authors:** Thomas Scheuerl, Meirion Hopkins, Reuben W. Nowell, Damian W. Rivett, Timothy G. Barraclough, Thomas Bell

**Affiliations:** 1 Department of Life Sciences, Imperial College London, Silwood Park Campus, Ascot, Berkshire, SL5 7PY UK; 2 Department of Natural Sciences, School of Science and Engineering, Manchester Metropolitan University, Manchester, UK; 3 Present Address: Department of Zoology, University of Oxford, 11a Mansfield Road, Oxford, OX1 3SZ UK

**Keywords:** Community ecology, Evolutionary ecology, Microbial ecology, Experimental evolution

Correction to: *Nature Communications* 10.1038/s41467-020-14570-z, published online 06 February 2020.

The original version of this Article contained an error in Fig. 1, in which the labels were inadvertently omitted from the pie chart. This has been corrected in both the PDF and HTML versions of the Article.

