# Supplementary material for: Bacterial adaptation is constrained in complex communities
Source: Nat Commun. 2020 Feb 6;11:754. doi: 10.1038/s41467-020-14570-z (PMC7005322; doi:10.1038/s41467-020-14570-z)
Supplement: Supplementary file 1 — Supplementary Information [file 41467_2020_14570_MOESM1_ESM.pdf]

Supplementary Information

Bacterial adaptation is constrained in complex communities

Scheuerl et al.

Supplementary Methods

Communities. We sampled water-filled tree-holes from the root buttresses of beech trees (*Fagus sylvatica*) between August 2013 and April 2014 from locations across the south of England. Tree-holes were sampled by homogenising the tree-hole by stirring prior to a 1 ml sample extracted and stored at ambient temperature while they were returned to the lab (<24 hours). Laboratory processing involved dilution 1:4 in sterile phosphate buffered saline (PBS) (pH 7.0) prior to filtration (pore size 20-22 µm, Whatman 4 filter paper). The filtrate was used to inoculate 5 ml sterile pH 7 beech leaf media (see Laboratory microcosms) and supplemented with 200 µg ml^{-1} cyclohexamide (Sigma). Communities were incubated at 22°C in static conditions for one week, to allow for communities to reach stationary phase, after which they were amended with a **freezing solution** of NaCl:glycerol (to a final concentration of 0.85% w/v: 30% v/v) and stored at -80°C. Communities were frozen so as to allow us to perform repeatable experiments using the same starting communities. Community composition was assessed using Illumina (250bp-paired end) sequencing (rtlgenomics.com), with operational taxonomic units (OTUs) specified at a 97% similarity cut-off. We standardised sequencing effort by randomly sampling 10,000 sequences from each sample. Diversity indices were calculated in R[1] using the *vegan* package[2]. We selected 11 communities that displayed a range of extrinsic properties (biodiversity, robustness) (see below).

Laboratory microcosms. All experiments and assays were conducted in deep 96-well microplates containing beech leaf media and incubated at 22°C unless stated otherwise. Beech leaf media (**BM**) was created by autoclaving 50 g of dried beech leaves in 500 ml of PBS[3], which gave a concentrated stock after filtration of coarse particles. pH 7 beech leaf media were produced by diluting this concentrated stock 32-fold in PBS. Our initial trials showed that bacteria grow slowly but for sustained periods in the media (weeks to months) even without media replacement (Supplementary Fig. 2). For this experiment, we altered the pH of the beech leaf medium by amending 10 ml of beech leaf media to 310 ml of deionised water and buffering with NaH_2PO_4= 2.5 g and Na_2HPO_4 =0.1 g, resulting in a pH of 5.5. Below, we refer to pH 7 and pH 5.5 BM as **BM7** and **BM5**.

Focal strains. We isolated bacteria from the 11 communities and allocated intrinsic properties to each isolate (see below). We selected 2 strains per community (22 in total) that displayed a range of intrinsic properties. We inoculated 50 µl of each frozen community into 1 ml BM7 and incubated the communities for 7 days. Fifty µl of the resulting communities were spread on R2A agar (Sigma-Aldrich, Gillingham, UK) at 22°C and after three days individual colonies were transferred to 1 ml BM7. After four days, the resulting liquid cultures were spread again on R2A plates as a second round for purification and a single colony was transferred to fresh BM7 and grown for seven days to maximum

density. The resulting monoclonal isolates were frozen at -80°C in freezing solution. Six of the focal isolates (*Serratia* sp., *Chryseobacterium*.1 sp., *Chryseobacterium*.2 sp., *Pantoea*.1 sp., *Pantoea*.2 sp. and *Bacillus* sp.) were obtained without growth in BM7 in order to obtain a broader range of phenotypes. Focal strains that were classified as the same genus were isolated from different communities. DNA was extracted from all of the isolates grown in BM7 using the ZR Fungal/Bacterial DNA Kit (Zymo Research, Irvine, CA, USA) following the manufacturer's protocol. A 1465 bp fragment of the 16S rRNA gene was amplified using RedTaq Ready Mix (Sigma-Aldrich) and the primer set 27f/1492r (Sigma-Aldrich) for sequencing by Macrogen (Amsterdam, Netherlands). PCR cycling parameters were as follows: 95 °C for 5 min, followed by 30 cycles of 30 s at 95 °C, 56 °C and 72 °C, with a final extension time of 7 min at 72 °C. 16S sequences of the focal strains were checked using `Chromas` program version 2.01, and aligned and trimmed in `ClustalX` program. R packages *ape*[4], *phangorn*[5] and *seqinr*[6] were used to represent the focal strains in a phylogenetic tree (Supplementary Fig. 3, see *Phylogenetic distance* below). After a likelihood ratio test, we calculated a distance matrix applying a Jukes-Cantor model. A neighbour-joining-tree was constructed using maximum likelihood and bootstrapping with a random seed and 1000 replications. We calculated the pairwise phylogenetic distance between all isolates to assess whether phylogenetically similar strains had similar evolutionary responses. Sequences were compared with type strains on BLAST (https://blast.ncbi.nlm.nih.gov/Blast.cgi) and the Ribosomal Database Project (http://rdp.cme.msu.edu/seqmatch/seqmatch_intro.jsp).

Evolution experiment summary. We allowed 22 focal strains to evolve in the presence of initially 11 experimental communities, or in the presence of a mixture of all of the isolates, or in the presence of themselves as a control. In the experiment, focal strains and communities were first grown up in BM7, before being transferred into BM5. Focal strains were then placed in dialysis bags (Pierce 96 well plate Thermo Scientific, MWCO 10 kDa) and suspended in communities inoculated into BM5. The focal strain-community pairs were tracked for 5 months. At the conclusion of the experiment, we assayed how the focal strains had changed relative to their ancestors (Supplementary Fig. 1). Details of the experiment follow. 3 background communities and the mixture of strains were excluded from subsequent analyses because of results indicating contamination into dialysis bags from the background community. Thus, we refer to 8 communities hereafter.

Preparation of focal strains. We first grew the 22 focal strains (and a mix of all isolates, and a sterile negative control) (n=4 replicates) in 900 µl BM7 for one week. We amended 400 µl of each culture with 320 µl freezing solution (as above) and stored the focal strains at -80°C. From the frozen samples, we inoculated 50 µl focal strains into 1.1 ml BM5 and grew them up for one week again. After that, 50 µl was subsampled into 500 µl of sterile deionised water and stored at -80°C after adding freezing solution. These samples were used as the `ancestors` in subsequent evolution assays.

Preparation of communities. We created a large stock of the communities by inoculating 50 µl of sample in 200 ml BM5 and incubating for seven days. This time period allowed for acclimatization to pH 5 conditions. These communities were then aliquoted into the experimental microcosms (1640 µl) to initiate the main experiment. Focal strains as background `community` were grown individually in BM5, without being exposed to biotic interaction, but allowing acclimatization to the environmental conditions.

Selection over time. Following the preliminary incubation steps, the ancestor focal strains were transferred into dialysis bags (Pierce 96 well plate Thermo Scientific, MWCO 10 kDa). The bags were sealed with aluminium foil strips and submerged in deep 96 well plates containing 1640 µl of background community. The dialysis bags were inoculated with 100 µl of one of the 22 focal strains, or a mix of the isolates, or a sterile control. The microcosms contained one of the communities or the ancestor focal species itself. Each focal strain x community treatment combination was replicated 4 times, resulting in 864 microcosms. The microcosms were incubated at 22°C for five months, covered in plastic bags to reduce evaporation. Every week, we replaced 10% of the medium (we removed 130 µl and added 164 µl which accounted for evaporation in the long term) in the microcosms with BM5. Unlike classic experimental evolution studies, this method does not maximise the number of generations, but we believe it is a more faithful reproduction of nutrient dynamics in most natural systems, which might be particularly important in experimental evolution studies that incorporate diverse communities.

We sampled the microcosms once per month: for background communities, a 125 µl sample was frozen at -80□C, and additional 5.55 µl were diluted in 49.45 µl of deionised water to track bacterial activity (Promega Bactiter-Glo, see section with functional measurements). The frozen samples were then used in the *Evolution assays* below. On each sampling day, we replenished the microcosms with 164 µl of fresh BM5 as above. The dialysis bags were sampled on the same day. For this we extracted 10 µl of sample, which was diluted into 100 µl of deionised water. The lost volume within the bags equilibrated by diffusion within 1 week. The 100 µl sample was split into two. First, 50 µl of the diluted sample were frozen at -80□C in 40 µl of freezing solution. The frozen samples were then used in the *Evolution assays* below. Second, 50 µl of diluted sample were transferred into a 96 white-well plate to read bacterial activity (Promega BactiterGlo). Due to the high workload, we separated the experiment into two blocks, which were sampled on different days. We refer to the focal strain sampled on the final timepoint as the 'Evolved' strain.

Evolution assays. We quantified adaptation of the focal strains by competing the evolved focal strain against their ancestor within the evolved community (Supplementary Fig. 1). We conducted the competition experiments by suspending two dialysis bags in a microcosm. One dialysis bag contained the evolved focal strain, the other bag contained the ancestral focal strain, and the microcosm contained the evolved community.

Experiment. We revived the evolved and ancestral focal strains by adding 40 µl of frozen sample to 860 µl BM5 and incubated for seven days. 100 µl of the evolved and the ancestor focal species were then dispensed into separate dialysis bags. In parallel we revived the evolved background community by inoculating 50 µl of each community into 5.5 ml BM5 in two deep 48-well plates. The 48-well plates were used to allow 2 dialysis bags into each well, allowing us to submerge both ancestral and evolved focal strains into the same community. The ancestor and evolved strains were incubated for 14 days. We recorded the population increase (cell counts), activity (data not presented here), and substrate degradation of the evolved and ancestral focal strains following incubation in the evolved communities on day 0, 7, and 14.

Cell counts and activity. We sampled 50 µl from the dialysis bags after 7 and 14 days of incubation (day 0 sample came from inoculation plate) and diluted the samples 1:3 in deionised water. 50 µl of the diluted sample were used to determine bacterial activity as above (Promega Bactiter-Glo). The results of the activity measurements will be presented in a separate paper. 10 µl of the diluted sample were further diluted in 90 µl of water containing 1% v/v (420 nM) thiazole orange (Sigma-Aldrich) and 1% v/v flow-cytometer beads (ThermoScientific). Cells were stained for 5 minutes, and were then counted using a BD Accuri C6 attached to an Intellicyt Hypercyt autosampler. Flow cytometry analysed 5 µl of the stained sample, and appropriate gating (SSC/FL1 533/30 nm, particles smaller than 8000 FSC-H were excluded) was designed to exclude non-fluorescent debris from the media. Particle that had fluorescence above 800 U was counted as bacterial cell. HyperCyt data files were analysed using in-house R[1] script based on the *flow-core* package[7]. The linear change over time for evolved/ancestor of each strain-community interaction was used as measurement of performance (see statistical details).

Substrate degradation. We measured the ability of the ancestral and evolved strains to degrade fluorescently-labelled substrates[8] (Sigma-Aldrich) to estimate the ecological niche. We used substrates that are common in plant leaf litter labelled with the fluorescent moiety 4-methylumbelliferone (MUB, Supplementary Table 2). Each of the substrates was incubated with a 25 µl sample to a final concentration of 40 µM[9] in filter sterilised water for 1 hour. After this time, 10 µl of 1 M sodium hydroxide was added and the fluorescence (Ex/Em:365 nm/445 nm) of the samples was measured immediately (Synergy HT; BioTek) over 4 minutes and the maximum value recorded.

Intrinsic factors. We characterised the focal strains according to their intrinsic properties:

i. Maladaptation. We assessed the degree of maladaptation of each focal strain and each community when the pH of the environment was altered to pH 5. Fifty µl of each community (4 replicates) or of each focal strain (3 replicates) was grown in 1640 µl of BM7 for 7 days. After this period, we perturbed the focal strains or communities by transferring a 50 µl sample to the new environment. The new environment altered the pH or salt concentrations (salt tested for communities only). Those media consisted of BM7 (control), pH 6 beech leaf media (320 ml BM6, 3.39 g NaH_2PO_4,

0.55 g Na_2HPO_4), pH 5.5 beech leaf media (320 ml BM5, 3.79 g NaH_2PO_4, 0.06 g Na_2HPO_4), or addition of 3.2 g or 1.6 g NaCl to 320 ml BM7. Bacterial activity was determined by recording the maximum luminescence (Synergy 2; BioTek) of 50 µl of sample mixed with 25 µl of Bactiter-Glo (Promega) over 6 minutes. Maladaptation was quantified as the mean difference in mean activity between the control environment (BM7) and the perturbed media (BM5) (Supplementary Fig. 4). The `maladaptation` (measured in the same way) of the experimental communities was taken to be an extrinsic factor. We refer to the extrinsic property as 'ecological robustness' and the intrinsic property as 'maladaptation' for clarity. Like for all other factors below, we also added the square of the factor to identify non-linear patterns in the data.

ii. Mean abundance. We used the 16S sequencing data from the initial communities to quantify the relative abundance of each focal strain across the communities. We used the genus-level names from the 16S sequencing data to identify the number of reads that corresponded to each of the focal strains.

iii. Genome size. We estimated genomes sizes from the whole-genome sequencing of the ancestral strains (see below).

iv. Phylogenetic distance. We used the phylogenetic tree of the focal strains (see Focal strains, Supplementary Fig. 3) to calculate the phylogenetic distance of every strain to *Raoultella* sp.2. *Raoultella* sp.2 was selected because it was one of the strains that clearly adapted during the experiment. Using the r package *ape* we to calculate the distance matrix using the 'JC69' model. This model was developed by Jukes and Cantor (1969)[10]. It assumes that all substitutions (i.e. a change of a base by another one) have the same probability. This probability is the same for all sites along the DNA sequence. Another assumption is that the base frequencies are balanced and thus equal to 0.25.

Extrinsic factors. The initial abiotic environment was the same across all microcosms, so the only extrinsic environmental influence on the evolution of the focal strains was due directly or indirectly to the communities in which they were suspended.

i. Biodiversity. We calculated diversity (Shannon's Index) based on the genus-level compositional data obtained by amplicon sequencing of the communities (see *Communities*). Shannon Index was defined as $H = -\sum_{i=1}^{S} p_i \ln(p_i)$, where p_i is the frequency of the ith genus and there are S genera in the community. We used the square of the Shannon Index (H^2) to identify non-linear patterns in the data.

ii. Robustness. The `ecological robustness` of the experimental communities was also used an extrinsic factor (see *Instrinsic factors - Maladaptation*).

Statistical analysis. Statistical analyses were conducted using R[1] following protocols given elsewhere[11,12].

Performance. We calculated the performance of each evolved strain as the evolved cell counts divided by the ancestral cell counts when they were competed over two weeks in the *Evolution assays*. We then assessed whether performance was associated with particular focal strains or communities (Fig. 1). Performance data (ratios of counts) were analysed using linear-mixed-effects models (LME) with `time` as non-linear continuous variable and an aggregation of `focal strain` and `community` as factorial variable (*nlme* package[13]). The models were calculated under restricted maximum likelihood, with `time` and `microcosm` entered as random effects. Model convergence was controlled using the *lmeControl* function, with the maximum number of iterations set to 500 and using optimisation *opt=optim* function[11]. We accounted for correlations of microcosms over time using a *corCompSymm* correlation structure[11] with `time` and `microcosms` as arguments. Divergence over time and associated change in heterogeneity were accounted for using a *varIdent* correction structure[11] with `time` and `focal strains` as arguments. We selected model parameters separately for each response variable (performance, enzyme production, activity, extrinsic-, intrinsic factors), simplifying the models using the Akaike`s Information Criterion (AIC). A repeated-measures-ANOVA with `time` and `microcosm` in the error term was used to explore contribution of `focal strain`, `community` and their `interaction` in the experiment (Fig. 1, Pie insert).

Extrinsic and intrinsic factors. We fitted LMEs to understand how extrinsic and intrinsic factors explained variation for measured trait data in a multiple linear regression approach (Fig. 2). Models for extrinsic and intrinsic variables were fitted separately and so we describe the general approach below. We first used the *corrgram* package[14] to check for variance inflation. Explanatory variables were centred around their mean to put them on a comparable scale and to account for variance inflation causing multicollinearity[15]. After that, we checked non-linearity by calculating generalized-additive-mixed-effect models GAMMs using the *mgcv* package[16] with cubic-regression and three knots. Finally, we constructed model trees of all variables to estimate the extent of interactions between explanatory variables using the *tree* package[17]. Based on those observations we fitted saturated or maximum LMEs with interactions between all main effects, as well as non-linear trends, following recipes for multiple-linear-regression given elsewhere[11,15,18]. To obtain correct degrees of freedom the error structure was constructed with `time` and either `focal strain` or `community` as arguments. Convergence control was applied whenever necessary. Models were corrected for heterogeneous variance structure in the residuals, which was mainly caused by Shannon`s index for communities and mean abundance for strains. For extrinsic factors, the background community `Isolate=Focal Strain` was removed for the analysis. The best fit model was selected using AIC. Models were fitted with the maximum likelihood and were simplified by stepwise deletion of non-significant terms using the *anova* function to compare models. When the simplified model was found, we refitted the model using the restricted maximum likelihood method. To analyse the change in enzyme use of the evolved to the ancestral value (Fig. 3), we used linear models with ancestral enzyme value the three different enzymes as explanatory variables.

Maladaptation and Robustness. Activity measurements for robustness of communities (square-root transformation) were compared using analyses of variance to test for differences between the environmental treatments of a specific population, followed by a Posthoc-TukeyHSD test (Supplementary Fig. 4). For maladaptation (log_{10} transformation) we used individual ANOVA tests between control and BM5. The estimate in mean difference was used as continuous variable for degree of maladaptation.

Re-sequencing of evolved communities. To assess changes in the background communities after 5 months, we 16S re-sequenced some of the final communities. For each of the 8 background communities, we selected the first two replicates containing bags for control (empty bag), the mix of strains, *Rizobium*.1 and N*ovosphingobium*.2 resulting in data for 64 background communities. Community composition of the frozen communities was assessed using Illumina MiSeq (250bp-paired end) sequencing performed by Molecular Research DNA (www.mrdnalab.com). The V4 region of the 16S rRNA gene was amplified, using primers 515f/806r with the forward primer barcoded. Sequences were curated using a propriety analysis pipeline by Molecular Research DNA; any sequences <150bp and those with ambiguous base calls removed, prior to denoising and editing for chimeras. Operational taxonomic units were specified at a 97% similarity cut-off. We randomly sampled 15,000 sequences per sample to normalise sequencing effort. We used principal coordinates ordination to visualise differences in the communities (function *cmdscale* in the *vegan* package[2]) using Bray-Curtis dissimilarity (*vegdist* function in *vegan* package). We assessed the significance of the treatments on OTU abundance using permutational analysis of variance implemented in the *adonis* function of the *vegan* package (Fig. 4).

Genomic changes. We conducted whole-genome sequencing of the focal strains *Raoultella* sp.1 and sp.2 across the different communities to detect genetic variants within the populations. We pooled 100 colonies collected from spread plates (R2A agar) of samples taken directly from the dialysis bags (3 days incubation). We also pooled 100 colonies from the ancestral populations that entered the dialysis bags (i.e. following the two weeks acclimatisation, Fig. S1). DNA extraction, library preparation, whole genome sequencing with minimum 30x fold coverage and *de novo* genome assembly was performed by MicrobesNG (Birmingham, United Kingdom, https://microbesng.uk/). Briefly, libraries were prepared using the Nextera XT Library Prep Kit (Illumina, San Diego, USA) and sequenced on an Illumina HiSeq 2500 using a 250bp paired end protocol. Raw data were filtered for adapter sequences and low quality bases using Trimmomatic v0.30[19], assembled using SPAdes v3.7[20], and the resulting scaffolds were annotated using PROKKA v1.11 3[21].

Potential contamination was checked by ascertaining the identity of the ancestral and evolved genomes during the course of the experiment. Scaffolds were queried against the NCBI non-redundant

nucleotide database (nt) using BLASTN[22] and plotted based on their %GC and read coverage using Blobtools v1.0[23,24]. Contaminants were identified by multiple clusters of scaffolds in GC-coverage space with different taxonomic annotations, indicating the presence of multiple species in the sample. Polymorphism in the ancestral cultures was ascertained by mapping these initial sequencing reads to the ancestral reference genome. Mapping and variant calling was performed using the BBMap v38.22 "variantPipeline.sh"[25]. Variants in the evolved samples were ascertained by mapping the evolved sequencing reads to the same reference, using the same approach. Ancestral and evolved variant calls were compared using RTG Tools v3.10.1 "vcfeval"[26] and sites showing a significant change in frequency in at least one sample compared to the ancestor were tabulated using custom R scripts. Proportion tests in R were used using counts of number of reads of each nucleotide variant between ancestor and evolved samples were used to determine significance of the change; using false discovery rate correction to control for multiple tests with the Benjamini–Hochberg procedure. Not all of the genomes were closed, so we may have missed some mutations. Ancestral genome sequences and derived SNP positions were visualised using the R package *BioCircos*[27].

Supplementary Figure 1. Schematic representation of the experimental design. The main features of the workflow for the evolution experiment are shown.

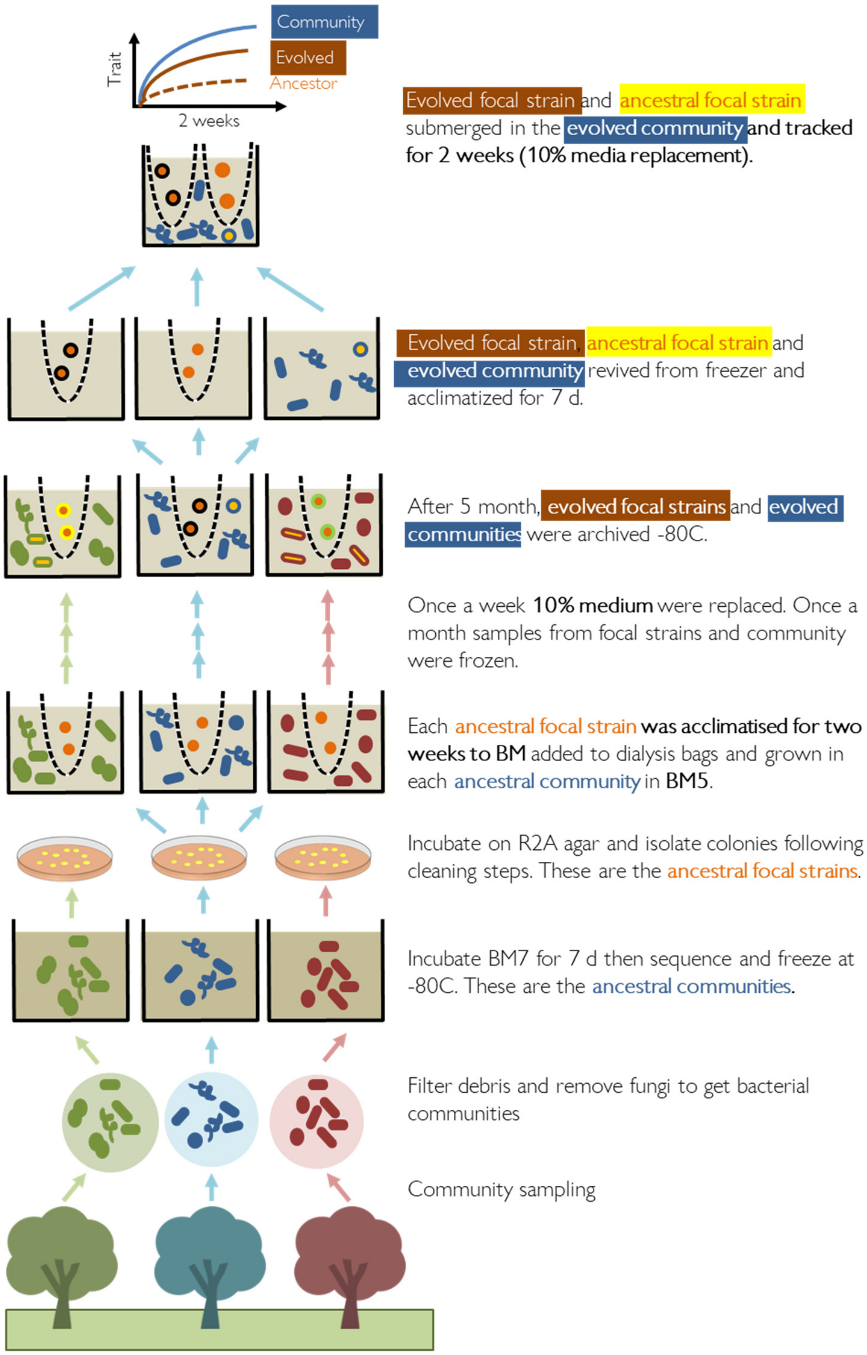

Supplementary Figure 2. Bacterial activity in BM7 over time. Populations were cultivated in BM7 without medium replacement. Two communities and two focal species were cultivated in 1640 µl BM7 for 23 days (n=4, 2 replicates from community 2 were sacrificed to get 2 control lines). For every population 12 microcosms were prepared and destructively sampled. Every second day 50 µl were sampled per microcosm and bacterial activity was measured. Even without replacement of resources, bacterial activity was high over this time period, suggesting that enough carbon resource is available in BM7 to sustain the population. Points and bars represent the means ± standard error. Source data are provided as a Source Data file.

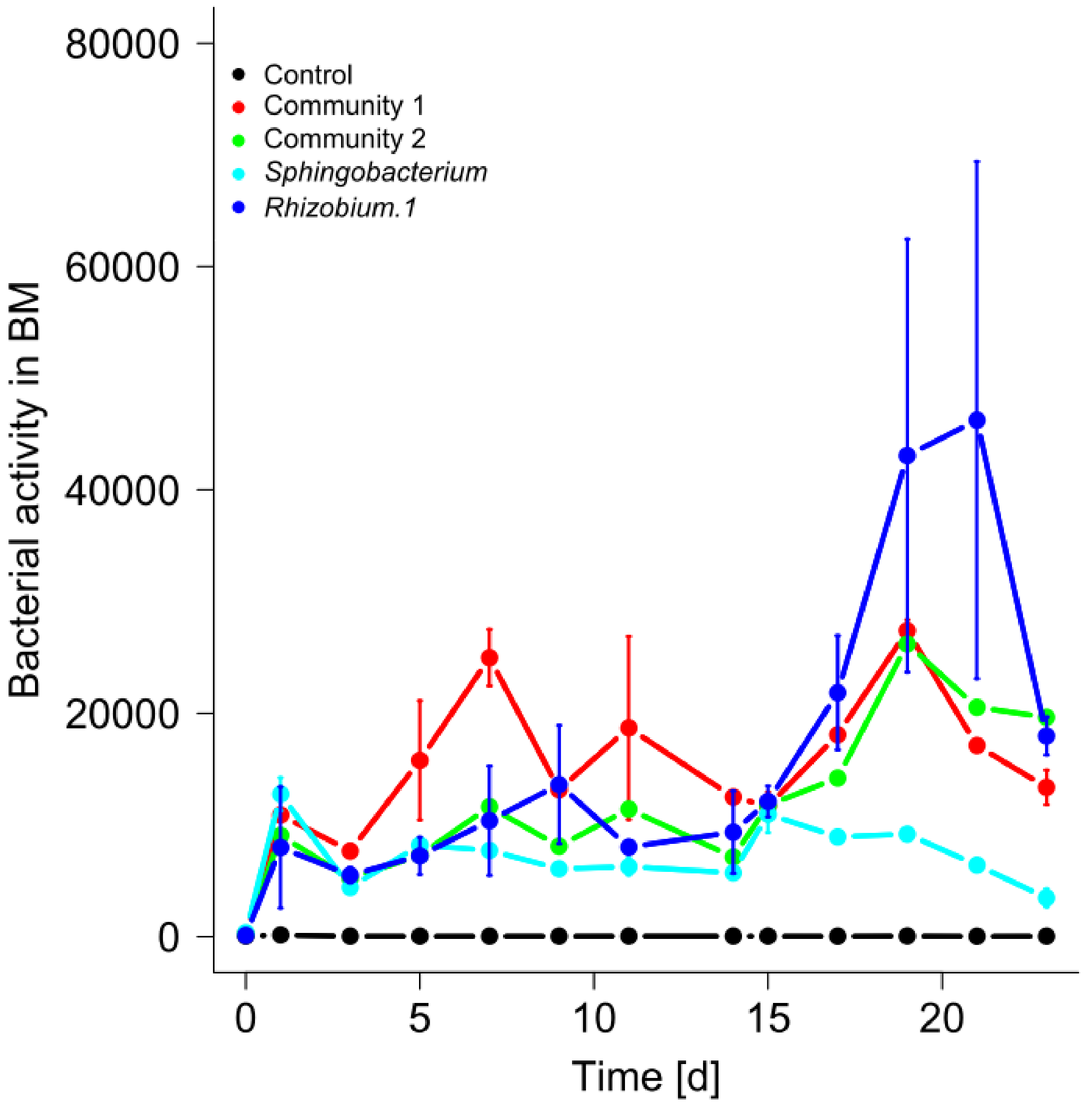

Supplementary Figure 3. Phylogeny of the focal strains. Sequences were aligned and trimmed, and a neighbour-joining tree was constructed on a distance matrix modelled by Jukes-Cantor. Maximum Likelihood was used, and bootstrapping was applied with a random seed and 1000 replications. After this, the tree was rooted with `Raoultella.2`, after which we calculated the phylogenetic distance of every species to `Raoultella.2`. Source data are provided as a Source Data file.

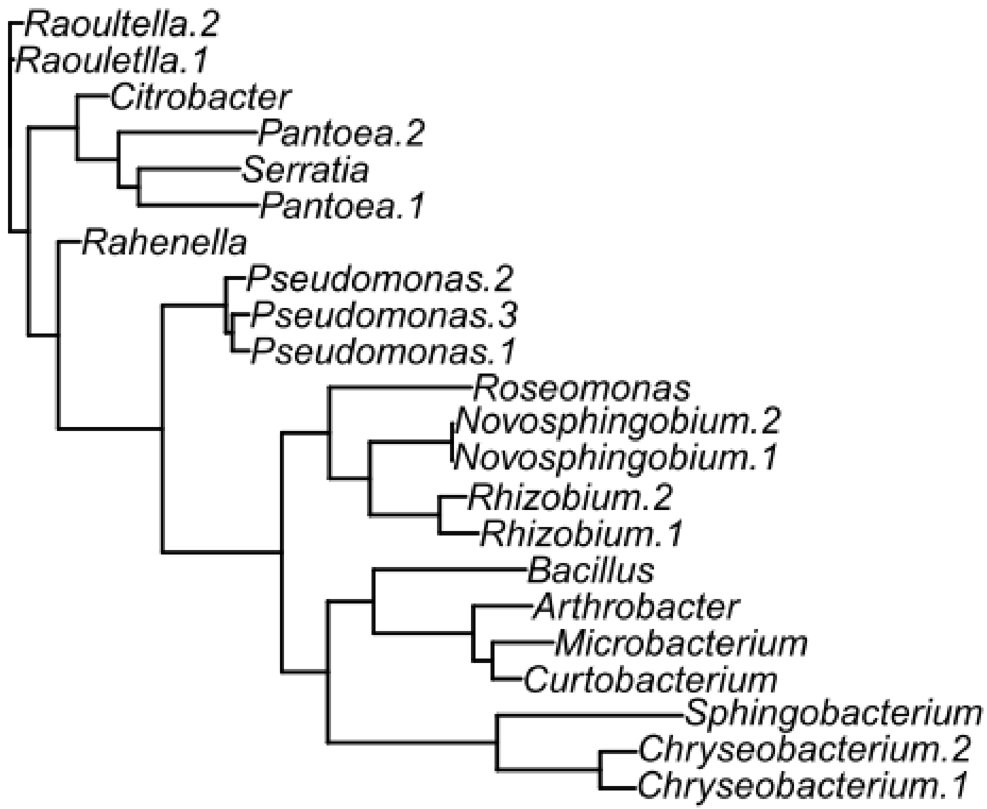

Supplementary Figure 4. Maladaptation of (a) communities and of (b) focal strains to media alteration. Control performances under BM7 were normalized to be zero and dots represent deviation of performance in BM5. Red dots represent significant growth alterations, while blue dots represent non-significant differences. **a** Performance of communities in BM5 compared to BM7. Individual community responses to treatment were compared using ANOVA tests after square-root transformation. **b** Performance of focal strains in BM5 compared to BM7. Individual strain responses to treatment were compared using ANOVA tests after log_{10} transformation. Horizontal line represents the mean growth in BM7. Source data are provided as a Source Data file.

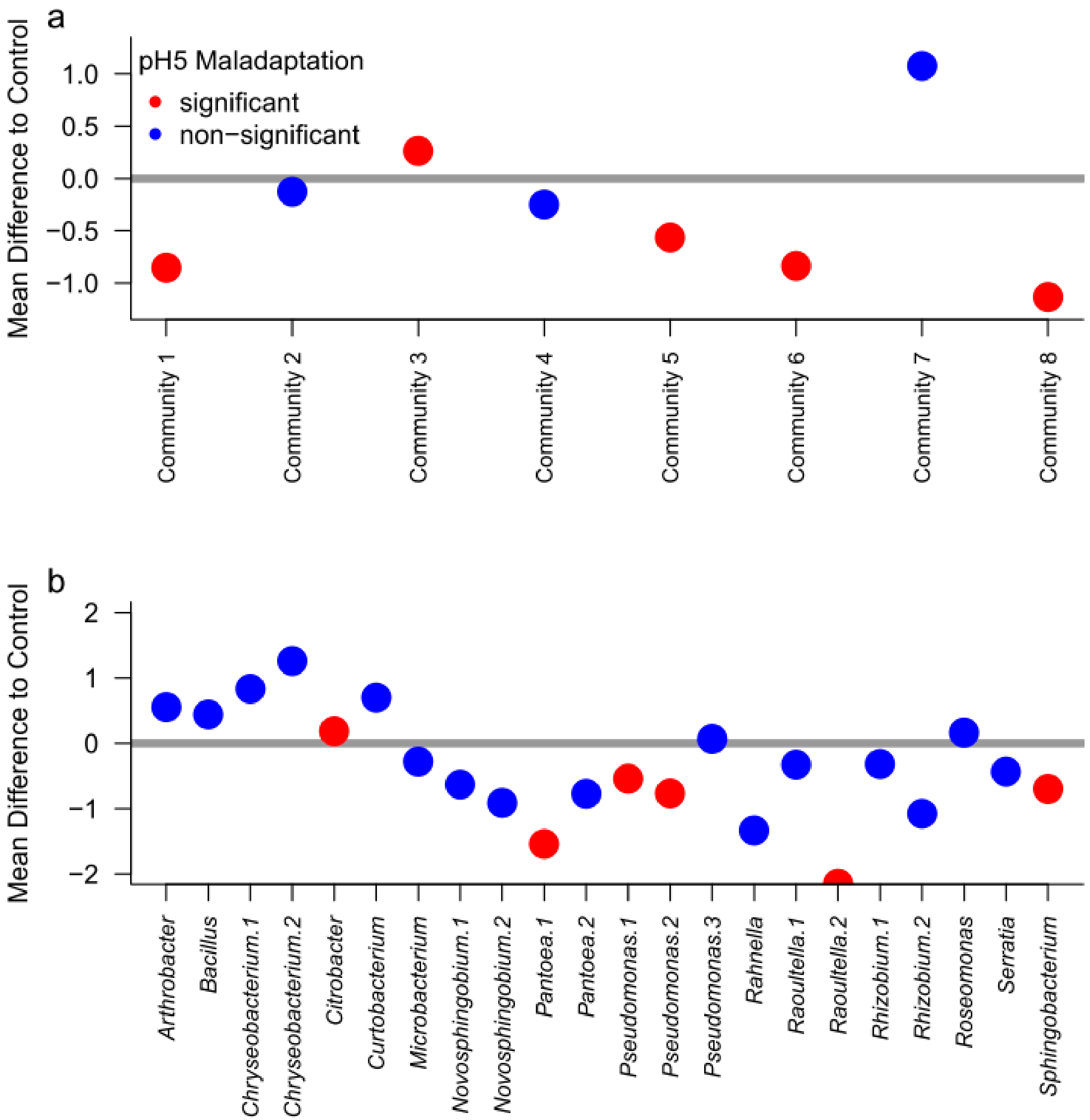

Supplementary Table 1. ANOVA explaining performance with focal strain and community

	Df	Sum Sq	Mean Sq	F value	Pr(>F)	
Focal Strain	23	33.07	1.44	9.38	0.0000	***
Community	8	31.92	3.99	26.04	0.0000	***
Strain x Community	184	96.17	0.52	3.41	0.0000	***
Residuals	648	99.29	0.15			

Signif. codes: 0 ‘***’ 0.001 ‘**’ 0.01 ‘*’ 0.05 ‘.’ 0.1 ‘ ’ 1

Supplementary Table 2. Substrate analogues used to assess extracellular enzyme activity.

Substrate	***Function***	***Enzyme***	***Nature of resource***
MUB-β-D-xylopyranoside	Hemicellulose degradation	Xylosidase	Labile
MUB-*N*-acetyl-β-D-glucosamine	β-1,4-glucosamine degradation	Chitinase	Intermediate
MUB-β-D-glucopyranoside	Cellulose degradation	B-glucosidase	Recalcitrant

Supplementary Table 3. Statistical correlations of response to selection from initial resource utilization.

	Estimate	Std. Error	t value	Pr(>|t|)	
(Intercept)	-9.8160	1.3750	-7.1370	0.0000	***
log10(ancestor)	16.5460	1.6280	10.1620	< 2e-16	***
Chitin	-1.6880	2.5890	-0.6520	0.5147	
Xylose	4.9740	2.2360	2.2240	0.0265	*
log10(ancestor):Chitin	1.6580	3.0730	0.5390	0.5897	
log10(ancestor):Xylose	-7.8040	2.4180	-3.2270	0.0013	**

Signif. codes: 0 '***' 0.001 '**' 0.01 '*' 0.05 '.' 0.1 ' ' 1

Residual standard error: 5.89 on 642 degrees of freedom

Multiple R-squared: 0.2151, Adjusted R-squared: 0.209

F-statistic: 35.19 on 5 and 642 DF, p-value: < 2.2e-16

Supplementary Table 4. Annotation of genetic variants.

Taxon	SNP	Location and annotation
Raoultella1	11_30225	Intergenic: 121bp upstream of oxyR_2; 131bp downstream of tRNA: oxidative stress sensor and regulator
	18_144490	kdgT gene: glycine to valine; 2-keto-3-deoxy-D-gluconate transporter; regulator of pectin metabolism
	27_3667	pipB2 gene: G to S; secreted effector protein
	27_3688	pipB2 gene: N to D
	27_3694	pipB2 gene: R to S
	27_3703	pipB2 gene: N to D
	64_1787	unannotated: non-genic
	13_123194	intergenic: 158bp upstream of livJ_2; Branched-chain amino acid ABC transporter substrate-binding protein
	13_8607	Coding gene: L to V; unannotated
	17_126931	Coding gene: G to D; unannotated
	23_21489	intergenic: 54bp downstream unannotated CDS
	31_4805	Coding gene: T to S; unannotated
	31_4806	Coding gene: T to R; unannotated
	31_4808	Coding gene: F to I; unannotated
	31_4809	Coding gene: F to C; unanotated
	31_4810	Coding gene: F to L; unannotated
	64_1723	unannotated: non-genic
Raoultella2	19_69325	Coding change: I to K; unannotated CDS
	7_315299	Intergenic: 70bp downstream fabG; fatty acid biosynthesis pathway
	18_10607	Silent change: narB; nitrogen reductase
	31_1063	23S-rRNA (BLAST ID)
		Coding: amino acid change
		Intergenic or silent change
		RNA

Supplementary References

1. R Development Core Team. The R project for statistical computing. Vienna, Austria: R Foundation for Statistical Computing. http://www.r-project.org/. *Vienna, Austria: R Foundation for Statistical Computing.* (2010). Available at: http://www.r-project.org/. (Accessed: 25th July 2012)
2. Oksanen, J. *et al. vegan: Community Ecology Package*. (2017).
3. Lawrence, D., Bell, T. & Barraclough, T. G. The Effect of Immigration on the Adaptation of Microbial Communities to Warming. *Am. Nat.* **187**, 236–248 (2016).
4. Paradis, E. *et al. ape: Analyses of Phylogenetics and Evolution*. (2017).
5. Schliep, K. *et al. phangorn: Phylogenetic Analysis in R*. (2017).
6. Charif, D. *et al. seqinr: Biological Sequences Retrieval and Analysis*. (2017).
7. flowCore. *Bioconductor* (2016). Available at: http://bioconductor.org/packages/flowCore/. (Accessed: 23rd December 2016)
8. Sinsabaugh, R. L., Antibus, R. K. & Linkins, A. E. enzymic approach to the analysis of microbial activity during plant litter decomposition. *Agric. Ecosyst. Environ.* (1991).
9. Frossard, A., Gerull, L., Mutz, M. & Gessner, M. O. Disconnect of microbial structure and function: enzyme activities and bacterial communities in nascent stream corridors. *ISME J.* **6**, 680–691 (2012).
10. Jukes, T. H. & Cantor, C. R. Evolution of protein molecules. in *Mammalian Protein Metabolism* (ed. Munro, H. N.) 21–132 (Academic Press, 1969). doi:10.1016/B978-1-4832-3211-9.50009-7
11. Zuur, A. F., Ieno, E. N., Walker, N., Saveliev, A. A. & Smith, G. M. *Mixed effects models and extensions in ecology with R*. (Springer, New York, 2009).
12. Scheuerl, T. & Stelzer, C.-P. Patterns and dynamics of rapid local adaptation and sex in varying habitat types in rotifers. *Ecol. Evol.* **3**, 4253–4264 (2013).

13. Pinheiro, J., Bates, D., DebRoy, S. & Sarkar, D. Linear and nonlinear mixed effects models CRAN - Package nlme. *http://cran.r-project.org/web/packages/nlme/index.html* (2012). Available at: http://cran.r-project.org/web/packages/nlme/index.html. (Accessed: 12th September 2012)
14. Wright, K. *corrgram: Plot a Correlogram*. (2017).
15. Logan, M. *Biostatistical design and analysis using R. A practical guide*. (John Wiley & Sons, 2010).
16. Woods, S. GAMs with GCV/AIC/REML smoothness estimation and GAMMs by PQL. R package version 1.7-9. *http://cran.r-project.org/web/packages/mgcv/index.html* (2012). Available at: http://cran.r-project.org/web/packages/mgcv/index.html. (Accessed: 12th September 2012)
17. Ripley, B. tree: Classification and Regression Trees. (2016). Available at: https://cran.r-project.org/web/packages/tree/index.html. (Accessed: 23rd December 2016)
18. Crawley, M. J. *The R Book*. (Wiley, 2012).
19. Bolger, A. M., Lohse, M. & Usadel, B. Trimmomatic: a flexible trimmer for Illumina sequence data. *Bioinforma. Oxf. Engl.* **30**, 2114–2120 (2014).
20. Bankevich, A. *et al.* SPAdes: a new genome assembly algorithm and its applications to single-cell sequencing. *J. Comput. Biol. J. Comput. Mol. Cell Biol.* **19**, 455–477 (2012).
21. Seemann, T. Prokka: rapid prokaryotic genome annotation. *Bioinforma. Oxf. Engl.* **30**, 2068–2069 (2014).
22. Altschul, S. F., Gish, W., Miller, W., Myers, E. W. & Lipman, D. J. Basic local alignment search tool. *J. Mol. Biol.* **215**, 403–410 (1990).
23. Kumar, S., Jones, M., Koutsovoulos, G., Clarke, M. & Blaxter, M. Blobology: exploring raw genome data for contaminants, symbionts and parasites using taxon-annotated GC-coverage plots. *Front. Genet.* **4**, 237 (2013).
24. Laetsch, D. R. & Blaxter, M. L. BlobTools: Interrogation of genome assemblies. *F1000Res* **6**, (2017).
25. Bushnell, B. BBTools. Available at: https://jgi.doe.gov/data-and-tools/bbtools/.

26. Cleary, J. G. *et al.* Comparing variant call Files for performance benchmarking of Next-Generation Sequencing variant calling pipelines. *bioRxiv* 023754 (2015). doi:10.1101/023754

27. Cui, Y. *et al.* BioCircos.js: an interactive Circos JavaScript library for biological data visualization on web applications. *Bioinformatics* **32**, 1740–1742 (2016).
