# Supplementary material for: Bacterial adaptation is constrained in complex communities
Source: Nat Commun. 2020 Feb 6;11:754. doi: 10.1038/s41467-020-14570-z (PMC7005322; doi:10.1038/s41467-020-14570-z)
Supplement: Supplementary file 3 — Reporting Summary [file 41467_2020_14570_MOESM3_ESM.pdf]

nature research

Corresponding author(s): Thomas Scheuerl

Last updated by author(s): YYYY-MM-DD

Reporting Summary

Nature Research wishes to improve the reproducibility of the work that we publish. This form provides structure for consistency and transparency in reporting. For further information on Nature Research policies, see Authors & Referees and the Editorial Policy Checklist .

Statistics

For all statistical analyses, confirm that the following items are present in the figure legend, table legend, main text, or Methods section.

n/a	Confirmed	
☐	☒	The exact sample size (n) for each experimental group/condition, given as a discrete number and unit of measurement
☐	☒	A statement on whether measurements were taken from distinct samples or whether the same sample was measured repeatedly
☐	☒	The statistical test(s) used AND whether they are one- or two-sided<br>*Only common tests should be described solely by name; describe more complex techniques in the Methods section.*
☐	☒	A description of all covariates tested
☐	☒	A description of any assumptions or corrections, such as tests of normality and adjustment for multiple comparisons
☐	☒	A full description of the statistical parameters including central tendency (e.g. means) or other basic estimates (e.g. regression coefficient) AND variation (e.g. standard deviation) or associated estimates of uncertainty (e.g. confidence intervals)
☐	☒	For null hypothesis testing, the test statistic (e.g. F, t, r) with confidence intervals, effect sizes, degrees of freedom and P value noted<br>*Give P values as exact values whenever suitable.*
☐	☒	For Bayesian analysis, information on the choice of priors and Markov chain Monte Carlo settings
☐	☒	For hierarchical and complex designs, identification of the appropriate level for tests and full reporting of outcomes
☐	☒	Estimates of effect sizes (e.g. Cohen's d, Pearson's r), indicating how they were calculated

Our web collection on statistics for biologists contains articles on many of the points above.

Software and code

Policy information about availability of computer code

Data collection	We used R for data representation
Data analysis	We used R for data analysis

For manuscripts utilizing custom algorithms or software that are central to the research but not yet described in published literature, software must be made available to editors/reviewers. We strongly encourage code deposition in a community repository (e.g. GitHub). See the Nature Research guidelines for submitting code & software for further information.

Data

Policy information about availability of data

All manuscripts must include a data availability statement. This statement should provide the following information, where applicable:

- Accession codes, unique identifiers, or web links for publicly available datasets
- A list of figures that have associated raw data
- A description of any restrictions on data availability

Manuscript contains a data availability statement.

Field-specific reporting

Please select the one below that is the best fit for your research. If you are not sure, read the appropriate sections before making your selection.

☐ Life sciences ☐ Behavioural & social sciences ☒ Ecological, evolutionary & environmental sciences

For a reference copy of the document with all sections, see nature.com/documents/nr-reporting-summary-flat.pdf

Life sciences study design

All studies must disclose on these points even when the disclosure is negative.

Sample size	We used 864 microcosms containing the 9 background communities and one of the 22 focal strains. We applied 4 replicates per focal strain community pair including a control and a strain mix
Data exclusions	No data were excluded for the analysis
Replication	We used four replicates per focal strain community combination
Randomization	We allocated samples randomly to the microcosms
Blinding	Samples were analysed using machines like flow cytometry or optical plate readers

Behavioural & social sciences study design

All studies must disclose on these points even when the disclosure is negative.

Study description	*Briefly describe the study type including whether data are quantitative, qualitative, or mixed-methods (e.g. qualitative cross-sectional, quantitative experimental, mixed-methods case study).*
Research sample	*State the research sample (e.g. Harvard university undergraduates, villagers in rural India) and provide relevant demographic information (e.g. age, sex) and indicate whether the sample is representative. Provide a rationale for the study sample chosen. For studies involving existing datasets, please describe the dataset and source.*
Sampling strategy	*Describe the sampling procedure (e.g. random, snowball, stratified, convenience). Describe the statistical methods that were used to predetermine sample size OR if no sample-size calculation was performed, describe how sample sizes were chosen and provide a rationale for why these sample sizes are sufficient. For qualitative data, please indicate whether data saturation was considered, and what criteria were used to decide that no further sampling was needed.*
Data collection	*Provide details about the data collection procedure, including the instruments or devices used to record the data (e.g. pen and paper, computer, eye tracker, video or audio equipment) whether anyone was present besides the participant(s) and the researcher, and whether the researcher was blind to experimental condition and/or the study hypothesis during data collection.*
Timing	*Indicate the start and stop dates of data collection. If there is a gap between collection periods, state the dates for each sample cohort.*
Data exclusions	*If no data were excluded from the analyses, state so OR if data were excluded, provide the exact number of exclusions and the rationale behind them, indicating whether exclusion criteria were pre-established.*
Non-participation	*State how many participants dropped out/declined participation and the reason(s) given OR provide response rate OR state that no participants dropped out/declined participation.*
Randomization	*If participants were not allocated into experimental groups, state so OR describe how participants were allocated to groups, and if allocation was not random, describe how covariates were controlled.*

Ecological, evolutionary & environmental sciences study design

All studies must disclose on these points even when the disclosure is negative.

Study description	We used 22 non pathogenic focal bacterial strains isolated from 11 beech tree communities. We then followed adaptive evolution of these focal strains while evolving for 6 month in the communities. At the end we compared the evolved strain and the ancestral strain while competing for resources in the coevolved background community for a period of two weeks.
Research sample	We used 864 microcosms containing the background community and one of the focal strains. We applied 4 replicates per focal strain community pair including a control and a strain mix.
Sampling strategy	For the evolved vs. ancestor comparison we sampled the initial time point, after 7 days and after 14 days.
Data collection	Data were collected using flow cytometry, optical density reading, genome sequencing and illumina sequencing.
Timing and spatial scale	The selection experiment ran for 6 month. The comparison between evolved and ancestral strain for two weeks.
Data exclusions	After the selection experiment we excluded 3 communities before we did the evolved/ancestor test (relevant for this study) as these indicated contamination.

Reproducibility	We included four replicates per focal strain community pair.
Randomization	Focal strains were randomly arranged in the microcosm plate.
Blinding	All smaples were analysed using machines like flow cytometry.

Did the study involve field work? ☐ Yes ☒ No

Field work, collection and transport

Field conditions	*Describe the study conditions for field work, providing relevant parameters (e.g. temperature, rainfall).*
Location	*State the location of the sampling or experiment, providing relevant parameters (e.g. latitude and longitude, elevation, water depth).*
Access and import/export	*Describe the efforts you have made to access habitats and to collect and import/export your samples in a responsible manner and in compliance with local, national and international laws, noting any permits that were obtained (give the name of the issuing authority, the date of issue, and any identifying information).*
Disturbance	*Describe any disturbance caused by the study and how it was minimized.*

Reporting for specific materials, systems and methods

We require information from authors about some types of materials, experimental systems and methods used in many studies. Here, indicate whether each material, system or method listed is relevant to your study. If you are not sure if a list item applies to your research, read the appropriate section before selecting a response.

Materials & experimental systems

n/a	Involved in the study
☐	☐ Antibodies
☐	☐ Eukaryotic cell lines
☐	☐ Palaeontology
☐	☐ Animals and other organisms
☐	☐ Human research participants
☐	☐ Clinical data

Methods

n/a	Involved in the study
☐	☐ ChIP-seq
☐	☒ Flow cytometry
☐	☐ MRI-based neuroimaging

Antibodies

Antibodies used	no antibiodies were used
Validation	not relevant

Eukaryotic cell lines

Policy information about cell lines

Cell line source(s)	not relevant
Authentication	not relevant
Mycoplasma contamination	not relevant
Commonly misidentified lines (See ICLAC register)	not relevant

Palaeontology

Specimen provenance	not relevant
Specimen deposition	not relevant
Dating methods	not relevant

☐ Tick this box to confirm that the raw and calibrated dates are available in the paper or in Supplementary Information.

Animals and other organisms

Policy information about studies involving animals; ARRIVE guidelines recommended for reporting animal research

Laboratory animals	not relevant
Wild animals	not relevant
Field-collected samples	Bacterial communities were sampled from natural sites located in UK
Ethics oversight	not relevant

Note that full information on the approval of the study protocol must also be provided in the manuscript.

Human research participants

Policy information about studies involving human research participants

Population characteristics	not relevant
Recruitment	not relevant
Ethics oversight	not relevant

Note that full information on the approval of the study protocol must also be provided in the manuscript.

Clinical data

Policy information about clinical studies

All manuscripts should comply with the ICMJEguidelines for publication of clinical research and a completedCONSORT checklist must be included with all submissions.

Clinical trial registration	not relevant
Study protocol	not relevant
Data collection	not relevant
Outcomes	not relevant

ChIP-seq

Data deposition

- [ ] Confirm that both raw and final processed data have been deposited in a public database such as GEO.
- [ ] Confirm that you have deposited or provided access to graph files (e.g. BED files) for the called peaks.

Data access links<br>*May remain private before publication.*	not relevant
Files in database submission	not relevant
Genome browser session<br>(e.g. UCSC)	not relevant

Methodology

Replicates	not relevant
Sequencing depth	not relevant
Antibodies	not relevant
Peak calling parameters	not relevant
Data quality	not relevant
Software	not relevant

Flow Cytometry

Plots

Confirm that:

- [x] The axis labels state the marker and fluorochrome used (e.g. CD4-FITC).
- [x] The axis scales are clearly visible. Include numbers along axes only for bottom left plot of group (a 'group' is an analysis of identical markers).
- [x] All plots are contour plots with outliers or pseudocolor plots.
- [x] A numerical value for number of cells or percentage (with statistics) is provided.

Methodology

Sample preparation	Samples were prepared according to the manufactures protocol. Bacterial cells were stained with thiazol orange.
Instrument	BD Accuri C6 attached to a Intellicyt Hypercyt autosampler
Software	HyperCyt data files were analysed using in-house R scripts
Cell population abundance	Whole population was considered
Gating strategy	According to the stained abcterial cells

- [x] Tick this box to confirm that a figure exemplifying the gating strategy is provided in the Supplementary Information.

Magnetic resonance imaging

Experimental design

Design type	not relevant
Design specifications	not relevant
Behavioral performance measures	not relevant

Acquisition

Imaging type(s)	not relevant
Field strength	not relevant
Sequence & imaging parameters	not relevant
Area of acquisition	not relevant

Diffusion MRI ☐ Used ☐ Not used

Preprocessing

Preprocessing software	not relevant
Normalization	not relevant
Normalization template	not relevant
Noise and artifact removal	not relevant
Volume censoring	not relevant

Statistical modeling & inference

Model type and settings	not relevant
Effect(s) tested	not relevant

Specify type of analysis: ☐ Whole brain ☐ ROI-based ☐ Both

Statistic type for inference
(See Eklund et al. 2016)

not relevant

Correction

not relevant

Models & analysis

n/a	Involved in the study
☒	☐ Functional and/or effective connectivity
☒	☐ Graph analysis
☒	☐ Multivariate modeling or predictive analysis

Functional and/or effective connectivity

not relevant

Graph analysis

not relevant

Multivariate modeling and predictive analysis

not relevant
